# Controlling Size in Multicellular Organs: Focus on the Leaf

**DOI:** 10.1371/journal.pbio.0060174

**Published:** 2008-07-15

**Authors:** Hirokazu Tsukaya

## Abstract

In leaves, cells get larger as cell division decreases or the ploidy increases. This might seem a logical response, but the controls are more complicated.

Leaf size depends on cell number and size. However, leaves are not simply the sum of cell size and number; rather, they are under the control of an unknown, organ-wide integration system. The existence of such a system is strongly suggested by two mysterious phenomena: compensation and high-ploidy syndrome. Compensation is characterized by cell enlargement triggered by a significant decrease in cellular proliferation, while plants with high-ploidy syndrome have more than eight sets of homologous chromosomes (8C), resulting in an increase in cell volume, but smaller leaves. Determining the mechanisms underlying these phenomena will provide important insight into the mechanism of multicellular organogenesis.

## What Drives Compensation?

In 1998, I noticed an unusual phenomenon in plants involving a decrease in the number of cells in the leaf lamina with a consequent increase in cell volume. I called this phenomenon “Hoshosayou,” which is Japanese for “compensation” [1], because I considered the inverse relationship (a decrease in cell number with an increase in cell volume) to be the most important feature, although the effect on leaf area was often incomplete. At that time, a limited number of examples were available to support the existence of such a relationship; however, additional evidence continues to accumulate. In 2002, having become convinced of the generality of the relationship, I adopted the English word “compensation” [2]. Gerrit Beemster of Ghent University (Belgium) also named this phenomenon compensation the following year [3]. We have since found that compensation occurs specifically in leaves and related lateral organs with a determinate fate (i.e., those organs that are under strict control in terms of when organogenesis starts and finishes), and not in indeterminate organs such as roots [4]. This likely reflects the presence of an unknown integration system that oversees cell division and expansion in determinate organs [5]. The details of this system are, at present, entirely unknown. Understanding the mechanism of multicellular organogenesis, many aspects of which remain mysterious, is one of the major goals of modern biology.

The mechanisms that integrate cellular proliferation with organ size in animals are also not yet understood [6,7]. As is widely known (even in Greek mythology), the size of the human liver is strictly controlled, and if most of the liver is surgically removed, it will regenerate to the proper size. Researchers have hypothesized a “total-mass checkpoint” mechanism(s) to explain this phenomenon [7], but, again, the molecular details are unknown.

Interestingly, several lines of evidence indicate that the mechanisms underlying the control of leaf size appear to differ from the total-mass checkpoint mechanisms. First, whereas a total-mass checkpoint in animals would prohibit organs larger than a standard size, leaves can become much larger than the standard size if cell division or expansion is enhanced [2,8–11]. Second, the trade-off-like relationship between number and size in leaf cells (i.e., compensation) is exhibited only when cell division is significantly decreased. However, a decrease in cell number does not always increase cell expansion [12–15], and such a response does not occur when cell expansion alone is altered [16–19]. A unidirectional relationship like this is unknown in animal organs. Thus, the question remains: what is the mechanism behind compensation?

## Compensation Rule Enigmas

When I talk about compensation at a seminar or symposium, nearly half of the audience responds by saying, “It should be a simple result of retarded cell division. If you cut a cake (or cell) in two, each piece is much larger than when you cut the cake (cell) in four.” In other words, they see compensation as a simple result of retarded division and not a unique phenomenon. This is not the case, however. The mechanisms involved in the coordination of cell growth and division are not simple, even in a budding yeast cell [20]. Let us examine some of the clues obtained from leaf development studies in the model plant Arabidopsis thaliana (referred to hereafter as Arabidopsis).

First, compensation cannot always “compensate” for the total mass or overall leaf shape and size. Arabidopsis plants over-expressing KIP-Related Protein 2 (KRP2) exhibit the typical features of compensation (smaller leaves containing fewer larger cells) [4,21], but they fail to maintain the overall size or shape of the leaves (Figure 1). Similarly, a series of mutants (the so-called *fugu* mutants) that typically exhibit compensation show significant variation in leaf size and shape [4].

**Figure 1:**
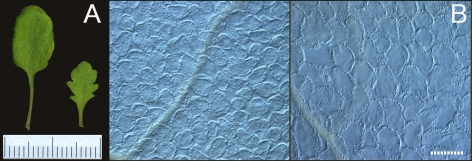
Compensation Does Not Maintain the Overall Size and Shape of Leaves (A) Gross morphology of the sixth leaf of wild-type Columbia (wt: left) and *KRP2*-over-expressor (o/x: right) plants. There are significant differences in the size and shape of the leaves. Scale bar, 1 mm. (B) A paradermal view of the first layer of palisade tissue from the leaves shown in panel (A): Left, wt Columbia, and right, *KRP2*-o/x. Scale bar, 100 μm. Note the increase in cell size (a result of compensated cell enlargement) in the *KPR2*-o/x.

Second, abnormally enhanced cell expansion (hereafter referred to as compensated cell enlargement) [4,19] is not observed during the developmental stage in which cell division actually occurs [4]. Compensated cell enlargement depends on a significant increase in the vacuolar volume (often ten times the cytoplasmic volume), and begins only after cell division ceases. Thus, compensation is not a passive result of decreased cell division (as in the cake analogy), but a result of unknown positive, emergent control mechanisms.

Third, compensation is not always observed when cell division declines in the leaf primordium. Large-scale screening of Arabidopsis leaf size/shape mutants found several mutants with a moderately low number of leaf cells but without compensated cell enlargement [12,13]. A reduction in cell number caused by the over-expression of the *BIG BROTHER* E3 ubiquitin ligase gene results in even smaller leaf cells [22]. Thus, compensation is not a simple trade-off between cell division and expansion in an organ of a given size.

Fourth, as mentioned above, changes in cell size are not observed when cell division is enhanced. For example, the loss-of-function mutation *ANGUSTIFOLIA3/GIF1* (*AN3/GIF1*) typically causes compensation (a shortened period of cell division that results in a decreased number of cells and increased cell expansion). However, over-expression of the same gene simply increases the period of cell division and the number of leaf cells without causing any abnormality in cell size [23]. A similar phenomenon has been reported for several other genes, including AINTEGUMENTA [24].

Fifth, we recently found that there may be a threshold decrease in cell division that triggers compensated cell enlargement (U. Fujikura, G. Horiguchi, and H. Tsukaya, unpublished data). Moreover, compensated cell enlargement is observed in the tip-most region of leaf primordia soon after the cells in the region stop dividing, whereas active cell division still occurs in the basal regions at this stage [4]. This fact strongly suggests that compensated cell enlargement is an expression of some emergent control of cell expansion that is not linked to the final number of cells in the primordium, but to some component (e.g., level or speed) of cellular proliferation. How, then, are they linked? Does the trigger for compensation function in a non-cell-autonomous or cell-autonomous manner? We do not yet know the answers to these questions, but a recent study revealed that only a subset of the genetic pathways involved in leaf-cell expansion, which are used in normal leaf growth, are enhanced when compensated cell enlargement is triggered [25]. This fact is an important clue as to the mechanism of compensation. The construction and analysis of chimeras for AN3 expression in leaf primordia should enhance our understanding of compensation, a big mystery that must be solved.

## High-Ploidy Syndrome Offers Clues to the Link between Ploidy and Organ Size

Another curious phenomenon in the organ-wide control of plant organ size is the link between ploidy and organ size. High-ploidy syndrome offers clues to this link. Colchicine-induced polyploidization has been widely used for horticultural improvement because tetraploid plants (described as 4C) are generally larger than the parental diploid (2C) plants. This effect on body size is attributable to increases in cell size [26]. Tetraploid Arabidopsis plants (4C = 20: four chromosomes of each type) are larger than their diploid parents (2C = 10) in terms of leaf, flower, and cell size in the leaf lamina (Figure 2).

**Figure 2:**
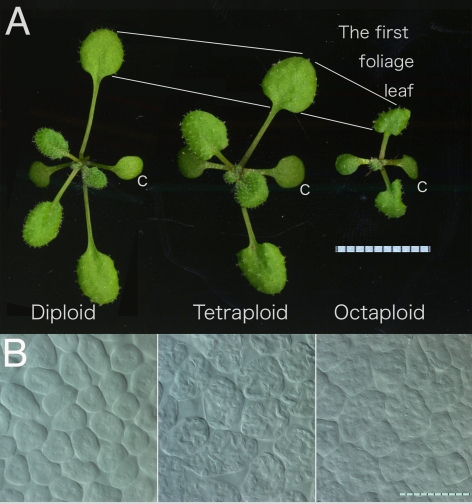
The Mysterious Relationship between Polyploidy and Organ/Cell Size Diploid, tetraploid, and octaploid Arabidopsis Columbia plants, two weeks after sowing, are shown from left to right. (A) Gross morphology of the plantlets. The octaploid plant has smaller leaves compared to the diploid and tetraploid plants. c, cotyledon. Unit of scale, 1 mm. (B) Paradermal view of the palisade cells in the first leaves. From left to right, diploid, tetraploid, and octaploid. Bar, 100 μm.

In addition, annual weeds such as Arabidopsis frequently exhibit repeated endoreduplication that results in cell-autonomous genome duplication during such developmental processes as trichome differentiation and increases in epidermal cell volume [27,28]. Endoreduplication is a modified version of the cell cycle that lacks M phase. During each round of endoreduplication, the genome is duplicated, but the number of chromosomes stays the same, resulting in polyteny (a condition in which the replicated chromosomes do not separate). Do these two methods of achieving polyploidy (i.e., increasing the number of chromosomes versus polyteny) work in the same way to increase cell volume?

We recently showed that tetraploidization partially rescues the cell-size defect in endoreduplication-deficient mutants, suggesting that cell size depends on genome size, regardless of the status of each chromosome (Figure 3) [29]. Thus, the answer seems to be yes, at least in part, although we do not know how ploidy and cell size are linked. A popular answer to this question is that increases in ploidy automatically and proportionally increase the amount of cellular components, resulting in larger cells. If this were true, cell volume after tetraploidization should be doubled, independent of mutations in cell size control. But fact refutes this prediction: the 4C/2C cell volume ratio varies among different strains (H. Tsukaya, unpublished data). Moreover, some reports have suggested that changes in ploidy do not affect cell size in particular organs [30]. Thus, why and how ploidy affects cell volume is entirely unknown [29].

**Figure 3:**
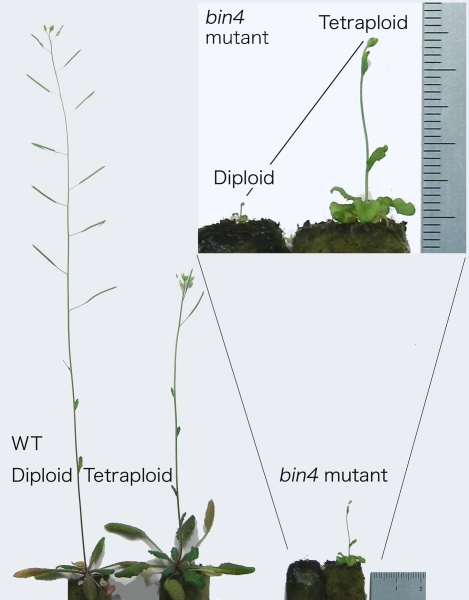
A Comparison of Diploid and Tetraploid Bin4 Mutant Plants to Diploid and Tetraploid Wild-Type Columbia Plants Tetraploidization partially recovered the dwarfism observed in the diploid *bin4* mutant. See [29] for further details. Unit of scale, 1 mm.

The relationship between ploidy and body/organ size is complex. As mentioned above, tetraploid plant organs/bodies are generally larger than diploid ones, and hexaploids are generally larger than tetraploids. However, octaploid plant organs/bodies are the same size as those in hexaploids, or sometimes much smaller than even those in diploids [31–33]; observations of octaploid Arabidopsis plants confirm this general rule (Figure 2). The leaf cells of octaploid plants are larger than those of tetraploids, whereas the leaves of octaploids are much smaller than those of tetraploids, suggesting that octaploids have fewer cells per leaf. Thus, higher ploidy enhances cell expansion but inhibits leaf cell division, leaving us with the “high-ploidy syndrome” dilemma.

This type of trade-off has long been known, even in animals [34]. Based on studies of 1C, 2C, 3C, 5C, and 7C salamanders, Fankhauser (1945) described the trade-off between cell volume and organ/body size as “compensation” [35]. For example, haploid salamanders have smaller cells than diploids, but the reduction is “completely compensated by [an] increase in cell number” [36]. Likewise, tetraploid salamanders have fewer but larger cells than do diploids [37]. A similar trade-off occurs in tetraploid mice [38]. The compensation observed in polyploid animals could be interpreted as the result of a strict total-mass checkpoint. Is this also true for the high-ploidy syndrome observed in plants? The answer is no because the presence of a total-mass checkpoint is not supported in plants, in which increases in cell volume are simply reflected in organ size (except for the above-mentioned cases in which severe decreases in cell division result in compensation) [2,9,11]. Is a total-mass checkpoint effective only in plants with a ploidy higher than 8C?

One possible explanation for high-ploidy syndrome is that at higher ploidy levels the cell cycle becomes more costly, so cell division slows [39], resulting in fewer than the expected number of cells and smaller organs. In fact, if tetraploid Arabidopsis seeds are sown together with diploid seeds, the tetraploids grow slightly more slowly in terms of the number of days to flowering and plastochron (see Figures 2 and 3), although the leaves and flowers of the mature tetraploids are much larger than those of the mature diploids. This explanation, however, contradicts the popular belief that increases in ploidy automatically and proportionally increase the amount of cellular components. If tetraploid cells are twice as rich as diploid cells in terms of their cellular components, the rate of cell growth should be the same in diploid and tetraploid cells. Why, then, do tetraploid organs expand more slowly than diploid ones?

One plausible answer is that an increase in ploidy alters the ratios between the various components of the cell with different dimensions, adding stress to cell activity [39]. For example, kinetochore attachment to the spindle pole body was altered in a tetraploid budding yeast in comparison to the diploid strain [40]. This alteration can be explained by an imbalance between the increase in surface area of the spindle pole body (bidimensional) and the length of the spindle (unidimensional). Even so, the answer above cannot explain why hexaploidy is the threshold for a proportional relationship between ploidy and organ size in plants. In addition, the length of the cell cycle is not altered by an increase in ploidy in unicellular budding yeast [40]. Apparently, there are rules that control organ size at the organ level; however, what these rules are remains, at present, an unsolved mystery.

How can we solve this mystery? The imbalance in mitosis in tetraploid budding yeast was identified from a genome-wide screen for mutants with ploidy-specific lethality [40]. Although Arabidopsis is not as suitable for the types of whole-genome-wide mutational approaches taken in budding yeast, mutational studies involving specific genes are easy. Moreover, leaves are produced repeatedly in plants and are easily analyzed; thus, leaves are a good model system for studies of the mechanisms underlying multicellular, determinate organ development. In fact, a series of tetraploid Arabidopsis mutants deduced from diploid single-gene mutants are being constructed in my laboratory, and have already yielded several clues as to the role of ploidy in cell/organ size control [29]. It is my hope that other researchers will focus on the mysteries surrounding the development of this simple but curious organ so that they may be resolved in the near future.

## Abbreviations

o/x: over-expressor; wt, wild-type
